# Prognostic role and clinicopathological features of SMAD4 gene mutation in colorectal cancer: a systematic review and meta-analysis

**DOI:** 10.1186/s12876-021-01864-9

**Published:** 2021-07-23

**Authors:** Tian Fang, Tingting Liang, Yizhuo Wang, Haitao Wu, Shuhan Liu, Linying Xie, Jiaying Liang, Chang Wang, Yehui Tan

**Affiliations:** Cancer Center, The First Hospital of Jilin University, No. 1 Xinmin Street, Changchun, 130021 Jilin Province China

**Keywords:** SMAD4, Gene mutation, Colorectal cancer, Prognosis, Meta-analysis

## Abstract

**Background:**

Approximately 5.0–24.2% of colorectal cancers (CRCs) have inactivating mutations in SMAD4, making it one of the frequently mutated genes in CRC. We thus carried out a comprehensive system review and meta-analysis investigating the prognostic significance and clinicopathological features of SMAD4 gene mutation in CRC patients.

**Methods:**

A detailed literature search was conducted in PubMed, Web of Science and Embase databases to study the relationship between SMAD4 mutations and the demographic and clinicopathological characteristics in CRC patients. The hazard ratios (HRs) with 95% confidence intervals (CI) were used to evaluate the effect of SMAD4 mutations on overall survival (OS) and progression-free survival (PFS)/recurrence-free survival (RFS).

**Results:**

Ten studies enrolling 4394 patients were eligible for inclusion. Data on OS were available from 5 studies and data on PFS/RFS were available from 3 studies. Comparing SMAD4-mutated CRC patients with SMAD4 wild-type CRC patients, the summary HR for OS was 1.46 (95% CI 1.28–1.67, *P* = 0.001), the summary HR for PFS/RFS was 1.59 (95% CI 1.14–2.22, *P* = 0.006). In terms of clinicopathology parameters, 9 studies have data that can be extracted, SMAD4 mutations were associated with tumor location (odds ratio [OR] = 1.15, colon/rectum, 95% CI 1.01–1.31, *P* = 0.042), TNM stage (OR = 1.28, stage IV/I–III, 95% CI 1.03–1.58, *P* = 0.025), lymph node metastasis (OR = 1.42, N1 + N2/N0, 95% CI 1.20–1.67, *P* < 0.001), mucinous differentiation (OR = 2.23, 95% CI 1.85–2.70, *P* < 0.001) and rat sarcoma viral oncogene homolog (RAS) mutation status (OR = 2.13, 95% CI 1.37–3.34, *P* = 0.001). No connection was found with age, gender, tumor grade, microsatellite instability status and b-viral oncogene homolog B1 mutation status. Besides, publication bias was not observed in any study.

**Conclusions:**

This meta-analysis suggests that SMAD4 mutation was associated with OS, PFS/RFS, and clinicopathological parameters, including tumor site, disease stage, RAS status, lymph node metastasis and mucinous differentiation. Our meta-analysis indicated that SMAD4 mutations could predict the poor prognosis and aggressive clinicopathological characteristics of CRC. More large-sample cohort studies are needed to confirm this conclusion. Since SMAD4 mutations are closely related to RAS mutations, their relationship warrants further investigation.

## Background

Colorectal cancer (CRC) is the third most common cancer and the second most common cause of cancer-related death over the world [1]. Despite advances in early diagnosis and treatment, lymphatic metastasis and distant metastasis are still the main causes of death in newly diagnosed CRC patients, and the overall survival (OS) rate of advanced CRC is still unsatisfactory.

The Cancer Genome Atlas database revealed that the mutation frequency of SMAD4 is 10%, which is one of the most common mutated genes in CRC [2]. SMAD4 is an established tumor suppressor gene located in chromosome band 18q21, and one of the most commonly destroyed gene in cancer among SMAD family genes [3]. This gene encodes a member of the Smad family of signal transduction proteins, that is phosphorylated and activated by transmembrane serine-threonine receptor kinases in response to transforming growth factor beta (TGF-β) signal transduction. The product of this gene forms homomeric complexes and heteromeric complexes with other activated Smad proteins in the context of activating by TGF-β receptors, then accumulate in the nucleus and regulate the transcription of target genes [4]. Mutations or deletions in the SMAD4 gene have been shown to result in pancreatic cancer [5], juvenile polyposis syndrome [6], and hereditary hemorrhagic telangiectasia [7]. In the past 2 decades, many studies had shown that SMAD4 mutation can not cause tumorigenesis by itself, but it can promote tumor progression caused by other genes [8]. The role of SMAD4 in CRC is similar to that in pancreatic cancer. The prevalence of SMAD4 mutations have recently been reported in 5.0–24.2% of several retrospective studies of sporadic CRC from 1999 to 2020 [9–14]. However, whether pathogenic mutation of SMAD4 reduces the OS in all CRC patients remains unclear. Therefore, we conducted a meta-analysis to assess the association of SMAD4 mutations with OS and PFS/RFS, as well as the relationship between SMAD4 mutations and clinicopathological characteristics of early and advanced CRC.

## Methods

### Search strategy

We conducted this study based on the preferred reporting items for Systematic Reviews and Meta-Analyses 2009 guidelines and registered with the International Prospective Register of Systematic Reviews, PROSPERO (identification code CRD42021244570). Systematic review of several databases was conducted in December 2020 with no lower limit set for date of publication. Search for related articles published in English or Chinese in the following electronic databases: PubMed, Web of Science, and Embase. The keywords “SMAD4” or “DPC4” and “colorectal cancer” or “colon cancer” or “rectum cancer” were used for relative articles searching.

### Study selection and inclusion criteria

All articles are limited to human studies published in English or Chinese that based on the following selection criteria: (1) Researches involved the prognostic of SMAD4 mutations in CRC patients, and provided sufficient information to obtain the Hazard ratios (HRs) and 95% confidence interval (CI) of OS or progression-free survival (PFS)/recurrence-free survival (RFS) directly or indirectly from the Kaplan–Meier curve. (2) Studies using surgical resection specimen of tumor to detect SMAD4 mutation in CRC. (3) The odds ratio (OR) associated with clinicopathologic features is given directly or can be obtained from computable data. (4) The study does not include CRC patients who received preoperative chemotherapy or radiotherapy. (5) Duplicate report results are unified by the latest or largest version.

### Data extraction and quality assessment

The two authors (F.T. and L.T) extracted all data sets from the selected studies independently, if there are any objections, we resolved through consensus or consultation with the corresponding author. The following information was collected from each study: first author, year of publication, country, time of diagnosis, sample size, CRC cases with SMAD4 gene mutations, sequencing methods of SMAD4 gene, mean follow-up periods and participants’ characteristics, including median age, gender, lymph node metastasis status, rat sarcoma viral oncogene homolog (RAS), b-viral oncogene homolog B1 (BRAF), microsatellite instability (MSI) status and mucinous differentiation as well as tumor stage. HRs and 95% CI of OS and PFS/RFS were extracted directly from papers, if not, we choose to extract from Kaplan–Meier Curve via Engauge Digitizer Version 4.1 (http://markummitchell.github.io/engauge-digitizer/). We used the Newcastle–Ottawa Scale to assess the methods and report quality of the included studies, and ranked them by score (8–9 points for high quality; 5–7 points for medium quality; less than 5 points for low quality) [15].

### Statistical analyses

HRs and ORs with their 95% CI were calculated. *P* value less than 0.05 was considered statistically significant. The Q statistic and I^2^ tests was used to estimate Heterogeneity among studies. The I^2^ statistic was ranged from 0 to 1. A random effect model was used for I^2^ > 0.5, which represented strong heterogeneity. Otherwise, fixed‐effect model would be applied. The analysis was performed to evaluate the impact of SMAD4 gene mutation on the prognosis of CRC. In addition, we evaluated the correlation between SMAD4 mutation status and different tumor grades, tumor differentiation, lymph node metastasis, and MSI/BRAF/RAS status. Sensitivity analysis was used to check data stability. The Egger’s test and Begg’s test were used for detection of publication bias, and *P* < 0.05 indicated significant bias. All analysis was performed with STATA 16.0 (Stata Corporation, College Station, TX, USA).

## Results

### Selection of studies

**Fig. 1:**
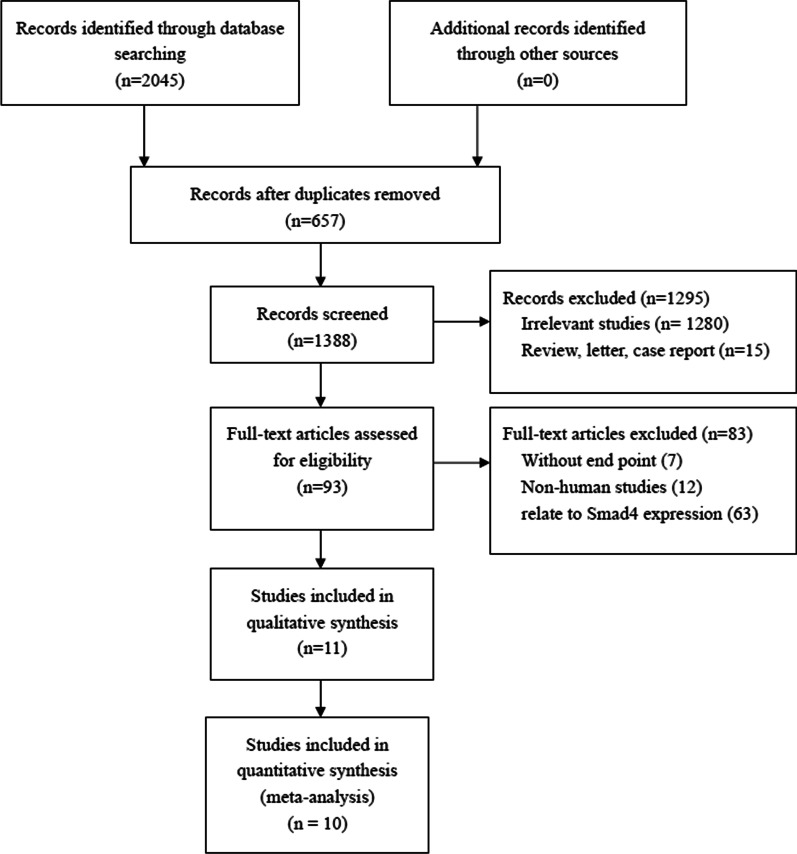
Schematic flow diagram for selection of included studies

The flowchart of the study selection is shown in Fig. 1. There were 465 articles identified from PubMed, 686 articles from Web of Science, 895 articles from Embase database. A total of 2045 articles were initially identified by the search strategy, and 657 full-text articles were retrieved after screening. Each selected article is tracked forward and backward, in case they contain another research of interest that has not yet been identified. Second, 1280 unrelated titles and abstracts were excluded from the study, and 108 full-text articles were evaluated for applicability, 7 articles were found to have no available outcome indicators or clinicopathologic features, 63 articles relate to SMAD4 protein expression and survival data, 12 articles were non-human trials and 15 articles were reviews, letters or case reports. A total of 4394 patients were included in the final ten studies [9–12, 16–21]. The detailed features of these articles are listed in Tables 1 and 2. The quality evaluation table of all articles is attached in Table 3.

**Table 1:** Main characteristics of studies included

Author	Year	Country	MA (year)	TNM stage	Time of diagnosis	MF (months)	Sample size	Sequencing methods	Mut: WT	Survival endpoints	HR(e)	NOS
Sarshekeh	2017	US	52	II–IV	2000–2014	50	734	HiSeq	90:644	OS; PFS	HR	9
Mizuno	2018	US	56	IV	2005–2015	22	237	NGS	31:206	OS RFS	HR Curve	9
Oyanagi	2019	Japan	NR	I–IV	2009–2015	NR	201	NGS	56:145	OS	Curve	7
Liao	2019	China	NR	I–IV	2013–2017	NR	84	NGS	1:2	NR	NR	8
Fleming	2013	Australia	69	I–IV	NR	NR	744	Applied Biosystems	64:680	NR	NR	6
Stahler	2020	Germany	NR	IV	2007–2012	40·3	373	NGS	NR	OS; PFS	HR	8
Jia	2017	US	NR	NR	NR	NR	53	NR	4:49	NR	NR	6
Ando	2005	Japan	66	A–D^a^	NR	NR	30	NR	1:29	NR	NR	7
Miyaki	1999	Japan	NR	NR	NR	NR	61	NR	9:52	NR	NR	6
Khan	2018	US	NR	NR	2012–2016	51.6	1877	NGS	226:1599	OS	HR	7

*NR* not report, *MA* mean age, *MF* median follow-up, *Mut* mutation, *WT* wild type, *HR* hazard ratio, *e* estimate, *NGS* next generation sequencing, *NOS* Newcastle–Ottawa scale, *OS* overall survival, *PFS* progression-free survival, *RFS* recurrence-free survival

^a^Dukes’stage

**Table 2:** Data extracted from studies included

Study	SMAD4 status	Age (years)		Gender		Location		Stage		Tumor grade		MSI status		RAS status		BRAF status		LN		Mucinous	
		< 65	≥ 65	Female	Male	Colon	Rectum	I–III	IV	WMD	PD	Stable	Unstable	WT	Mut	WT	Mut	Yes	No	Yes	No
Sarshekeh 2017	Mut	75	15	52	38	75	15	6	74	50	13	NR		NR		NR		NR		NR	
	WT	561	83	286	358	410	234	85	526	357	114										
Mizuno 2018	Mut	NR		17	20	28	9	NR		34	3	NR		10	27	34	3	31	6	NR	
	WT			110	131	218	60			214	27			135	106	238	3	148	93		
Oyanagi 2019	Mut	32	24	19	37	NR	NR	18	38	NR		NR		NR		52	4	NR		17	11
	WT	68	77	65	80			72	73							136	9			9	48
Liao 2019	Mut	NR		11	17	15	8	16	6	NR		20	2	6	22	26	2	16	6	NR	
	WT			30	26	34	23	53	3			40	14	27	29	45	11	26	30		
Fleming 2013	Mut	NR		34	30	53	11	52	12	48	13	58	6	NR		NR		NR		24	38
	WT			296	384	501	178	593	87	498	158	588	92							133	540
Jia 2017	Mut	NR		NR		NR		3	1	NR		NR		NR		NR		NR		NR	
	WT							41	8												
Ando 2005	Mut	0	1	0	1	1	0	1	0	1	0	NR		NR		NR		NR		NR	
	WT	12	17	9	20	15	14	22	7	29	0										
Miyaki 1999	Mut	NR		NR		NR		3	6	NR		NR		NR		NR		NR		NR	
	WT							41	11												
Khan 2018	Mut	NR		NR		NR		NR		NR		NR		NR		NR		NR		65	161
	WT																			212	1387
Stahler 2020	Mut	NR		NR		NR		NR		NR		NR		NR		NR		NR		NR	
	WT																				

*Mut* mutated, *WT* wild type, *WMD* well to moderately differentiated, *PD* poorly differentiated, *LN* lymph node metastases, *NR* not reported

**Table 3:** Quality assessment according to the Newcastle–Ottawa scale of the included studies

Author	Selection	Comparability	Exposure	Total score
Sarshekeh 2017	4	2	3	9
Mizuno 2018	4	2	3	9
Oyanagi 2019	3	2	2	7
Liao 2019	4	2	2	8
Fleming 2013	3	1	2	6
Jia 2017	3	2	3	8
Ando 2005	3	1	2	6
Miyaki 1999	3	2	2	7
Khan 2018	3	1	2	6
Stahler 2020	3	2	3	7

### Relationship between SMAD4 mutations and CRC prognosis

**Fig. 2:**
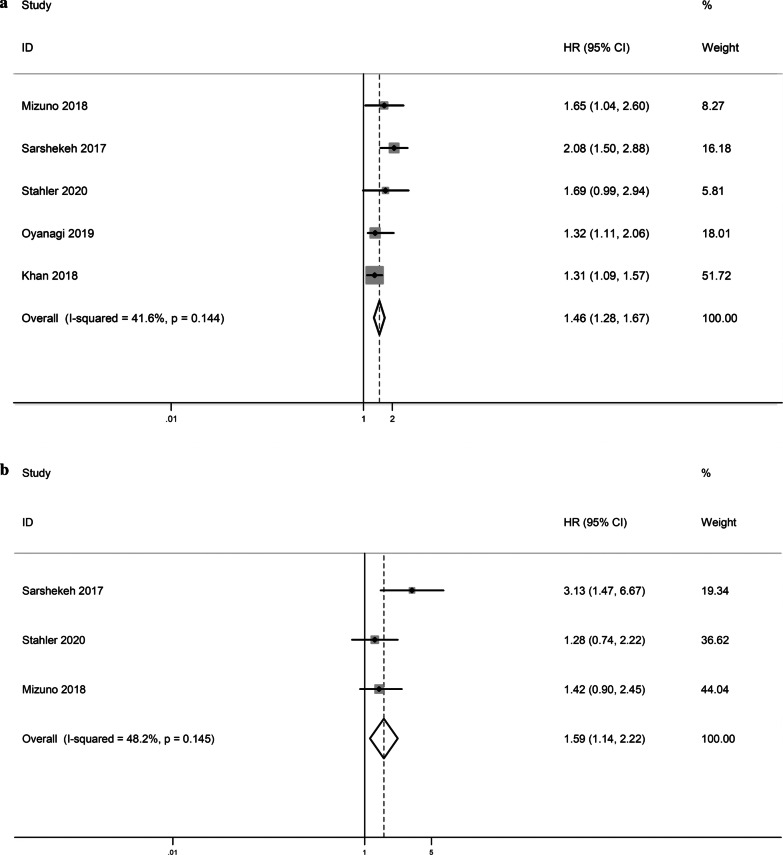
**a** Forest plot for meta-analysis of the association of SMAD4 mutations with OS of patients with CRC. **b** Forest plot for meta-analysis of the association of SMAD4 mutations with PFS/RFS of patients with CRC

A total of 5 articles provided OS related data. Due to the moderate heterogeneity (I^2^ = 41.6%, P heterogeneity = 0.144), we use the fixed-effect model to pool HR. Comparing SMAD4 mutant patients with SMAD4 wild-type patients in CRC, the summary HR for OS was 1.46 (95% CI 1.28–1.67, *P* = 0.001) (Fig. 2a).

A total of 3 articles provided PFS/RFS related data. Comparing SMAD4 mutant patients with SMAD4 wild-type patients in CRC, the summary HR for PFS/RFS was 1.59 (95% CI 1.14–2.22, *P* = 0.006) (Fig. 2b) and there was moderate heterogeneity between the studies (I^2^ = 48.2%, P heterogeneity = 0.145), so we use the fixed-effect model to pool HR.

### Relationship between SMAD4 mutations and clinicopathologic features of CRC

A total of 9 studies have data that can be extracted from clinicopathologic results, the specific characteristics of which are detailed in Table 2. The clinicopathologic OR values of the final merger are presented in Table 4. In terms of clinicopathology parameters, SMAD4 mutations were associated with tumor location (OR = 1.15, for colon versus rectum, 95% CI 1.01–1.31, *P* = 0.042), pathological TNM stage (OR = 1.28, for stage IV vs I–III, 95% CI 1.03–1.58, *P* = 0.025), lymph node metastasis (OR = 1.42, for N1 + N2 vs N0, 95% CI 1.20–1.67, *P* < 0.001), mucinous differentiation (OR = 2.23, 95% CI 1.85–2.70, *P* < 0.001) and RAS mutations (OR = 2.13, 95% CI 1.37–3.34, *P* = 0.001). However, SMAD4 gene mutation has no connection with other clinicopathology parameters, including patient age, gender, tumor grade, MSI status and BRAF status.

**Table 4:** Relationship of SMAD4 gene and clinicopathologic characteristics of colorectal cancer

Features	Experimental group	Control group	OR (95% CI)	*P* value
Age (years)	< 65	≥ 65	1.01 (0.91, 1.12)	0.854
Gender	Female	Male	1.09 (0.95, 1.24)	0.212
Tumor location	Colon	Rectum	1.15 (1.01, 1.31)	0.042
TNM stage	IV	I–III	1.28 (1.03, 1.58)	0.025
Tumor grade	Well to moderate	Poor	1.04 (0.96, 1.12)	0.318
MSI	Stable	Unstable	1.10 (0.95, 1.28)	0.191
RAS	Mut	WT	2.13 (1.37, 3.34)	0.001
BRAF	Mut	WT	1.00 (0.90,1.09)	0.976
Lymph node metastasis	N1 + N2	N0	1.42 (1.20,1.67)	0.000
Mucinous differentiation	Mucinous	Other	2.23 (1.85,2.70)	0.000

*MSI* microsatellite instability, *RAS* rat sarcoma viral oncogene homolog, *BRAF* b-viral oncogene homolog B1

### Sensitivity analysis and publication bias

**Fig. 3:**
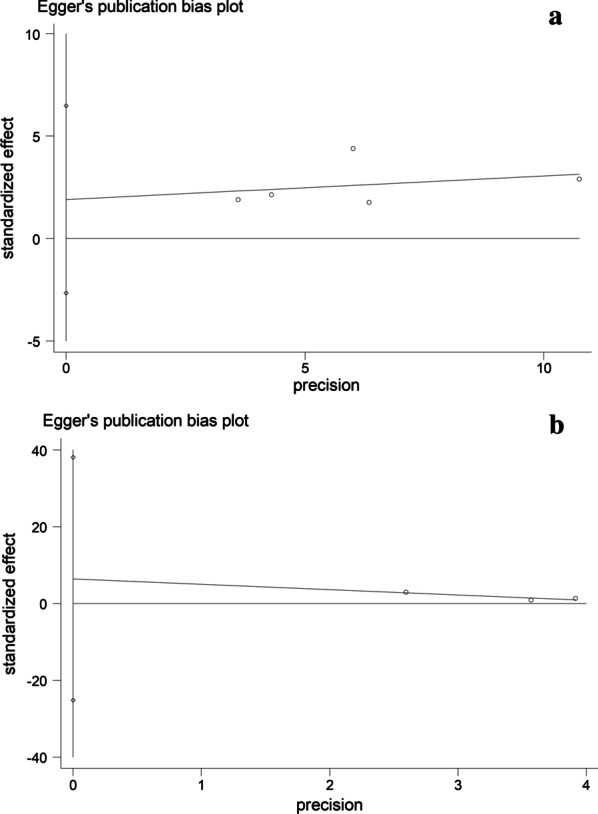
**a** Forest plot of Egger’s test for publication bias of OS. **b** Forest plot of Egger’s test for publication bias of PFS/RFS

**Fig. 4:**
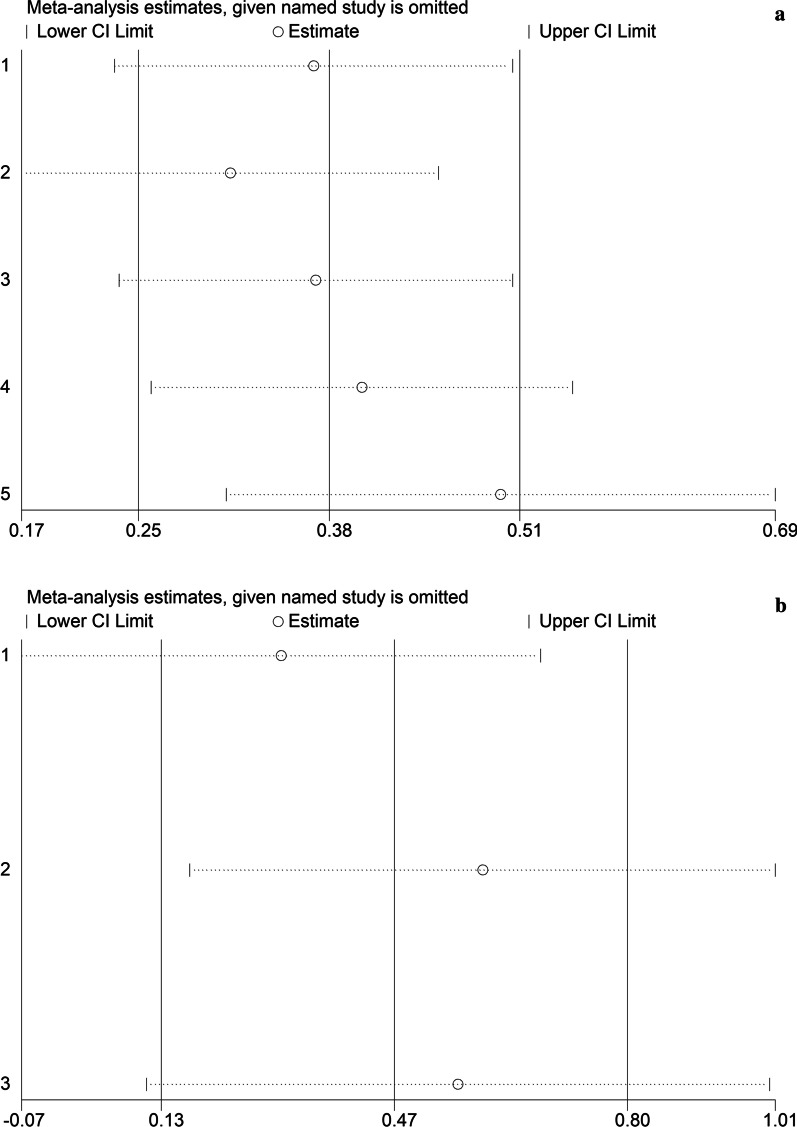
**a** Sensitivity analysis of meta-analysis of the association of SMAD4 mutations with OS in CRC patients. **b** Sensitivity analysis of meta-analysis of the association of SMAD4 mutations with PFS/RFS in CRC patients

Our analysis of publication bias using correlation test revealed that there is no obvious publication bias for OS (*P* = 0.277 for Begg’s test and 0.221 for Egger's test) (Fig. 3a) and PFS/RFS (*P* = 0.235 for Begg's test and 1.000 for Egger's test) (Fig. 3b). In addition, the sensitivity analysis confirmed that the results were reliable for OS (Fig. 4a) and PFS/RFS (Fig. 4b).

## Discussion

The role of Smad4 mutations of the prognosis and clinicopathological parameters in CRC has been investigated in several studies, but the results are inconsistent. In addition, no meta-analysis has been conducted to evaluate the impact of SMAD4 gene on the prognosis of CRC. Therefore, we conducted a meta-analysis and suggested that SMAD4 pathogenic mutations were associated with poor prognosis in CRC. Compared with the SMAD4 wild-type controls, SMAD4 mutations are associated with worse OS (pooled HR = 1.46, 95% CI 1.28–1.67, *P* < 0.001) and worse PFS/RFS (HR = 1.59, 95% CI 1.14–2.22, *P* = 0.006). In order to further investigate the role of SMAD4 gene in CRC, we also analyzed the relationship between SMAD4 status with clinical pathological parameters of CRC, the results show that patients with SMAD4 mutations have higher pathological TNM stages (stage IV/I–III; pooled OR = 1.28; 95% CI 1.03–1.58), that is, distant metastasis is more likely to occur in patients with SMAD4 mutations. And SMAD4 mutant patients were more likely to feature mucinous differentiation (pooled OR = 2.23; 95%CI 1.85–2.70, *P* = 0.000), tumors are more likely to occur in the colon (pooled OR = 1.15; 95% CI 1.01–1.31; *P* = 0.042), more prone to lymph node metastasis (N1 + N2/N0; pooled OR = 1.42; 95% CI 1.20–1.67; *P* = 0.000), and to harbor concurrent RAS mutations (pooled OR = 2.13; 95% CI 1.37–3.34; *P* = 0.001). Importantly, all of these parameters generally indicate a poor prognosis. Combined OR suggested that SMAD4 gene mutation has nothing to do with age, gender, tumor grade, MSI or BRAF status. The effect of SMAD4 gene on MSI or BRAF status remains to be elucidated. Another meta-analysis [14] showed that SMAD4-mutated patients were at a higher risk of distant metastasis (combined OR 2.04, 95% CI 1.41–2.95), which is consistent with our results.

Over the past 2 decades, many studies have shown that SMAD4 mutation does not cause tumorigenesis by itself, but it can promote tumor progression caused by other genes [8]. Ohtaki et al [22]*.* reported that the frequency of SMAD4 mutations were significantly higher in tumors with liver metastasis than in those without such metastasis. Inamoto et al*.* [23] reported that SMAD4-deficient colorectal tumor cells secreted more CCL9 and CCL15, these two chemokines recruit CCR1 + myeloid cells through CCL9-CCR1 and CCL15-CCR1 axis, resulting in metastasis. Vauthey et al*.* [24] concluded that patients with SMAD4 mutations are less likely to undergo repeated hepatectomy due to recurrent disease after the initial tumor resection. Alhopuro et al*.* [25] showed that SMAD4 is a predictive biomarker for 5-fluorouracil (5-Fu) based chemotherapy in CRC patients. Zhang et al. [26] discovered a novel mechanism mediated by SMAD4 to trigger 5-Fu chemosensitivity through cell cycle arrest by inhibiting the PI3K/Akt/CDC2/survivin cascade. Mei et al*.* [27] suggested that SMAD4 mutations could be potential biomarkers for poor prognosis of cetuximab-based therapy, which needs to be further validated in a larger patient cohort. Lin et al*.* [28] found that silencing SMAD4 reduces the sensitivity of CRC cells to cetuximab by promoting epithelial-mesenchymal transition (EMT), while the high expression of Smad4 may be clinically beneficial to cetuximab-based therapy. Mizuno et al*.* [11] found that SMAD4 mutation was significantly associated with poor OS following hepatic resection, which was independent of RAS mutation status. These findings indicate that SMAD4 pathogenic variants play a key role in tumor progression and the efficacy of target therapy in CRC patients.

In the current analysis, researchers found that SMAD4 gene alteration was significantly associated with loss of SMAD4 expression in CRC, and loss of SMAD4 disrupts canonical TGF-β signaling [29], because it is a signaling transcription factor. In addition, it is reported that the loss of SMAD4 function is independently associated with the reduction of RFS and OS in CRC patients, especially patients with advanced disease [30]. In contrast, CRC patients with high Smad4 expression had a much longer median OS than those with low Smad4 expression [31]. Germline mutations of TGF-β family signaling pathway genes significantly increase the risk of having colonic neoplasia [32]. The canonical TGF-β/Smad4 signaling pathway acts as a tumor suppressor in early stages, which is characterized by its anti-proliferative activity, ability to induce apoptosis and promote genome stability, while TGF-β acts as a metastasis promoter to stimulate the development of advanced tumors [33].

EMT is a well-coordinated process in which epithelial cells lose cell connectivity and polarity and transform into mesenchymal cells with migration and invasion capabilities. Studies have suggested that EMT is a key step in tumor progression and metastasis, and the TGF-β1 signaling plays a key role in EMT [34]. Functional study results indicate that TGF-β-induced Smad4-dependent EMT followed by apoptosis in CRC cells [35, 36]. Siraj et al*.* [37] identified TGF-β-induced EMT was insufficient to obtain invasive potential, while the activated RAS would alter the reaction, imparting tumorigenic and invasive potential. Therefore, the synergistic effect between Ras-Raf-MAPK and TGF-β/Smad cascades is a necessary condition for the acquisition of aggressive phenotype in cancer.

At present, RAS has been recognized as tumor driver gene, predictive biomarker and therapeutic target in CRC. The expression of RAS up-regulates the expression of phosphotyrosine kinase receptors ERBB1 (EGFR) and ERBB2 (HER2) and induces an aggressive phenotype. Smad4-dependent signal transduction negatively regulates the expression of these receptors and inhibits Ras-induced upregulation of EGFR and ERBB2, thus exerting an antiproliferative effect. The loss of oncogenic RAS and SMAD4 signals synergistically upregulate the abnormal expression of EGFR and ERBB2, leading to the development of neoplasm and the metastasis and spread of the primary tumor [38, 39]. TGF-β can quickly activated RAS and ERK pathway [40], in contrast, the ERK pathway inhibits the TGF-β/Smad4 pathway by phosphorylating Smad2 and Smad3 at serine or threonine residues in the linker region, so epithelial cells with oncogenic RAS mutations usually exhibit loss of TGF-β antiproliferative response [8]. Patients with RAS wild-type tumors and retained SMAD4 wild-type had longer OS than patients with both mutations [41]. However, SMAD4 mutations were significantly associated with poorer OS regardless of RAS mutation status or other clinicopathological factors. The precise cooperative mechanisms of SMAD4 with other genes of influence also requires further examination.

Given the relative frequency of SMAD4 mutations in CRC patients, routine SMAD4 testing may be appropriate. For individualized treatment of CRC, SMAD4, as a driver mutation, will become a novel target for precision medical treatment of CRC, and further research should be done for guiding clinical decision-making.

No heterogeneity or publication bias was found in this meta-analysis, and sensitivity analysis shows that our results are reliable. However, this analysis has several limitations. First, our meta-analysis included studies of qualified articles published in English or Chinese, and did not include relevant articles written in other languages or unpublished papers, which is likely to result in selection bias. Second, the use of specific therapies and tumor stage differed among the included articles. Third, the HR calculated from the data or extracted from the survival curve may not be as reliable as the HR calculated directly using the analysis of variance. Therefore, the results should be carefully interpreted. However, as far as we know, this is the first meta-analysis to demonstrate SMAD4 mutation by evaluating the pathological features and prognostication in CRC.

## Conclusion

In conclusion, we found that SMAD4 mutation was associated with poor prognosis in CRC, but has nothing to do with MSI status, BRAF status or tumor grade. Further studies are needed to evaluate these findings and the clinical significance of SMAD4 status in CRC.

## Data Availability

All data generated or analyzed during this study are included in this article.

## Abbreviations

CRC: Colorectal cancer
OS: Overall survival
TGF-β: Transforming growth factor beta
HRs: Hazard ratios
ORs: Odds ratios
CI: Confidence interval
RAS: Rat sarcoma viral oncogene homolog
BRAF: B-viral oncogene homolog B1
MSI: Microsatellite instability
PFS: Progression-free survival
RFS: Recurrence-free survival
5-Fu: 5-Fluorouracil
EMT: Epithelial-mesenchymal transition
